# Hospital Administration

**Published:** 1896-12-12

**Authors:** 


### The Institutional Workshop.

### HOSPITAL ADMINISTRATION.

### THE LONDON HOSPITAL.

The Hon. Sydney Holland in the Chair.

Mr. Hale, who has given a great deal of time to the duties of the office during the last few years, has resigned the chairmanship of the committee of this hospital, and the Hon. Sydney Holland has been appointed to that office. Mr. Hale, the late chairman, displayed the utmost interest in the welfare of the hospital in every way, and has given an immense amount of time to the work, in which he took the keenest interest. He is entitled to the grateful acknowledgments not only of his colleagues, but of the whole body of Governors, for his services as chairman.

The Hospital has stood by the London during its times of adversity, and has been the means of strengthening the hands of the executive and of helping it materially on several occasions. We may therefore exercise a friend's privilege by speaking with frankness of the new situation created by the Hon. Sydney Holland's acceptance of office. The London Hospital experienced a serious loss by the retirement of Mr. Nixon, the house-governor, whose knowledge, administrative ability, and devotion to the best interests of the charity, left a void on his retirement which has not yet been filled. For some years most of the energy and all the initiative have been supplied by the matron. The post of matron at this hospital is so laborious that if its occupant were to be strictly confined to the discharge of its duties they would be found more than enough to tax the strength of the majority of women. It is certainly most desirable that the administration of a great institution like the London Hospital should be controlled by a strong man possessing the full confidence and support of an active and vigorous committee, full of zeal for the best interests of so noble an institution. Therefore we welcome the Hon. Sydney Holland's appointment. His record shows him to be a man capable of doing for the London Hospital all that it has so long needed, if it is to maintain its proper position among the great medical institutions of this country. He has worked assiduously to make himself acquainted with the best methods of hospital administration, and his work at the Poplar Hospital proves him to be thorough in practice in the best meaning of that word. We hope, however, that Mr. Holland will concentrate his energies for a whole year, at any rate, upon the work of the London Hospital, for otherwise, despite his energy, the reforms which have been so long needed must still be delayed.

It is no easy task which lies before the new chairman of the London Hospital. He will find that the whole administration needs overhauling, that the executive require a strong hand over them to enforce necessary discipline among the higher officials, some of whom have the character of being exceptionally severe in their attitude towards the humbler members of the resident staff. To keep each official in his or her proper place, to persuade those who may have assumed to themselves an authority not warranted by the regulations under which they act, and to secure that everybody in the institution shall be made thoroughly happy by being treated with absolute justice and fairness, is no light work for the new chairman to undertake. Yet this, and much more, is required if the London Hospital is to take a high place for efficiency amongst the great metropolitan charities; and there is, besides, the necessity of reducing the terrible strain which has been placed upon the nursing staff of the London Hospital for years by the unjustifiable practices which have crept in under the old taking-in rules. We were told quite recently by one of the most distinguished members of the medical profession, who is a trained hospital administrator, that week after week he has seen a large number of cases admitted under this or that member of the honorary medical staff to wards which were already overcrowded. This system, which has recently been modified, should engage Mr. Holland's close attention, for it is the true cause of the enfeebled condition into which many members of the nursing staff were shown to have fallen in the course of the inquiry before the House of Lords. Another question involving a considerable expenditure, which Mr. Holland appreciates, we believe, is the imperative necessity to refurnish a large number of the wards, and to introduce modern appliances and fittings. The commissariat should also be overhauled, and we are confident Mr. Holland will speedily abolish the present practice of compelling the in-patients, or most of them, to supply themselves with tea, sugar, and butter, which certainly should not find a place in a hospital locker.

We hope that Mr. Holland may be able to induce young, active, and intelligent men to join the committee as vacancies occur, and to devote their time and strength to the London Hospital. If the completeness and efficiency which Mr. Holland has brought to the Poplar Hospital may be taken, as in our opinion it certainly may, as an indication of his influence for good, then the new chairman of the London Hospital will render a service to the greatest of the voluntary hospitals which should win for him a considerable reputation, and make him one of the most important and valuable of men in the hospital world. In any case, we wish the Hon. Sydney Holland a brilliant success, and we are confident that whilst he will certainly impress his personality upon the whole institution and enforce the necessary discipline, he will at the same time so administer the affairs of the London Hospital that no one will have any just cause of complaint, whilst everybody will be led to make the welfare and progress of the institution their first consideration.

### THE GREAT NORTHERN CENTRAL HOSPITAL.

Mrs. Harkness and the Ladies' Committee.

The Great Northern Central Hospital owes much of its prosperity and success to the ladies of North London. They have been indefatigable in their efforts to help the funds, and those efforts have been marked by continuity and persistence extending over a number of years, a fact which proves that the authorities of Charing Cross Hospital and of every metropolitan hospital would be wise to enlist the services of the ladies by organising a similar effort through them to increase the annual income. The life and soul of the Ladies' Committee of the Great Northern Central Hospital is Mrs. Harkness, who has displayed an energy, a kindliness, a self-denying devotion, and a tact on all occasions which have won for her the esteem and admiration of all who have a knowledge of the facts. We are glad to note that the views here expressed are those of the supporters of the Great Northern Central Hospital, who have combined to present Mrs. Harkness with a silver tea service suitably engraved. Good wine needs no bush, and good work like Mrs. Harkness' needs no publicity, for the subscriptions to this presentation fund have been raised quite privately, and we know that, had people been aware that such a movement was on foot, the sum actually received would have been twice the amount it actually reached. All these facts combine to encourage similar efforts on similar lines on behalf of the hospitals all over the country.

